# Long-Term Outcomes of a Self-Expanding Transcatheter Heart Valve

**DOI:** 10.1016/j.shj.2026.100841

**Published:** 2026-04-04

**Authors:** Won-Keun Kim, Dimytri Siqueira, Helge Möllmann, Oliver Dörr, Matthias Renker, Efstratios Charitos, Stephanie Brunner, Yeong-Hoon Choi, Samuel Sossalla, Holger Nef, Thomas Walther, Alexandre Abizaid, Stefan Toggweiler

**Affiliations:** a Department of Cardiology, Justus-Liebig University of Giessen, Giessen, Germany; b Kerckhoff Heart Center, Department of Cardiology, Bad Nauheim, Germany; c DZHK (German Centre for Cardiovascular Research), Giessen, Germany; d Department of Cardiology, Instituto Dante Pazzanese de Cardiologia, São Paulo, Brazil; e St. Johannes Hospital, Department of Cardiology, Dortmund, Germany; f Cardioangiological Center Bethanien, Frankfurt, Germany; g Kerckhoff Heart Center, Department of Cardiac Surgery, Bad Nauheim, Germany; h Heart Center Lucerne, Department of Cardiology, Lucerne, Switzerland; i Heart Center, Segeberger Kliniken, Bad Segeberg, Germany; j Johann-Wolfgang-Goethe Universität, Department of Cardiac Surgery, Frankfurt, Germany; k Heart Institute (InCor), University of São Paulo Medical School, São Paulo, Brazil

**Keywords:** Degeneration, Durability, Long-term outcome, Structural valve deterioration

## Abstract

**Background:**

Data on long-term outcomes and durability of the ACURATE neo valve are limited. This study aimed to obtain long-term outcome and echocardiographic data among ACURATE neo recipients.

**Methods:**

Consecutive patients undergoing transfemoral transcatheter aortic valve implantation for severe native aortic stenosis from 4 centers using the ACURATE neo valve between 2012 and 2018 (n = 961) were included. Exclusion criteria for the durability analysis were 30-day mortality, valve-in-valve implantation or surgical aortic valve replacement within 30 days, and lack of echocardiographic follow-up (FU) beyond 30 days, leaving 758 patients included in the durability cohort. Primary endpoints were the cumulative incidence of late bioprosthetic valve failure (BVF), structural valve deterioration (SVD), and Kaplan–Meier estimates of all-cause mortality.

**Results:**

In the overall cohort (median age of 82 years, 61.3% females, median and maximum FU time of 5.0 and 11.3 years), estimates of all-cause mortality at 1, 5, and 8 years were 13.6, 43.1, and 67.3%, respectively. In the durability cohort, the cumulative incidence was 3.5% for moderate SVD, 2.1% for severe SVD, and 3.2% for BVF at 8 years. Paired FU echocardiography beyond 5 years (n = 243, median FU time of 6.4 years) showed mean aortic valve gradients and effective orifice area of 9 mmHg and 1.8 cm^2^ post-transcatheter aortic valve implantation and 6 mmHg and 1.7 cm^2^ at final FU echo.

**Conclusions:**

In this multicenter observational cohort treated with the ACURATE neo platform, 8-year survival was 32.7%, and the cumulative incidence of severe SVD (2.1%) and BVF (3.2%) was limited. These findings provide long-term durability estimates in an elderly, real-world population.

## Highlights

• The Kaplan–Meier estimate of all-cause mortality at 8 years was 67.3%.
• The cumulative incidence at 8 years was 2.1% for severe structural valve deterioration and 3.2% for bioprosthetic valve failure.
• Valvular aortic regurgitation was the most common mode of failure.
• Long-term valve performance after a median of 6.4 years was preserved.
• These findings may be helpful in the management of patients that were treated with this device.

## Graphical abstract

Graphical Abstract: **ACURATE neo long-term outcomes.** Mortality was analyzed in the survival cohort (n = 961), SVD and BVF were assessed in the durability cohort (n = 758), and valve performance ≥5 years was determined in a subset of patients with paired echocardiography follow up exceeding 5 years (n = 243). Abbreviations: AS, aortic stenosis; BVF, bioprosthetic valve failure; DP mean, mean transaortic gradient; EOA, effective orifice area; SVD, structural valve deterioration; TF, transfemoral.

**Figure:**
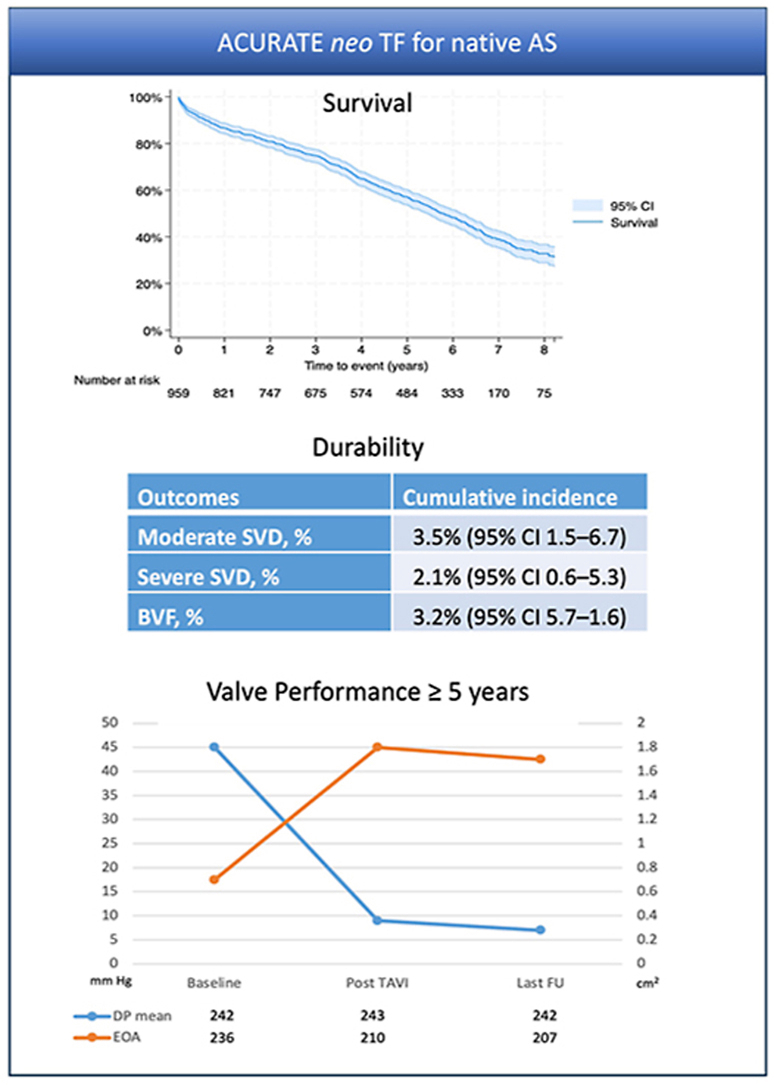

## Introduction

Transcatheter aortic valve implantation (TAVI) has evolved as the standard treatment option for patients with severe aortic stenosis. Among elderly patients, TAVI is indicated irrespective of the operative risk, whereas in younger patients, TAVI may be considered in higher-risk situations. According to European guidelines, the age threshold for TAVI irrespective of operative risk is 75 years.^1^ In recent years, outcomes have improved substantially, and there is already a shift toward the treatment of younger and lower-risk cohorts, even though this trend is not supported due to the lack of long-term durability data of transcatheter heart valves (THVs). Current data on long-term THV performance have several limitations, including follow-up (FU) periods rarely exceeding 5 years, incomplete serial echocardiographic valve assessment, relatively small sample sizes, and the competing risk of death among elderly study patients and its appropriate statistical appraisal. The history of surgical bioprostheses suggests that structural valve deterioration (SVD) or bioprosthetic valve failure (BVF) as clinical correlate may increasingly occur beyond a period of 5 years.^2^ Finally, long-term data are not available for all THV types, and in the absence of a class effect, long-term data of a specific THV type may not necessarily be extrapolated to others.

The ACURATE platform (neo, neo2, and Prime) represents a self-expanding valve with supra-annular design that is characterized by low gradients, low rates of conduction disturbances, low risk of coronary obstruction, and easy coronary access.^3^^,^^4^ The first iteration received CE mark in 2011, but so far only midterm echocardiographic FU data were available.^5^ Following unfavorable outcomes in the Investigational Device Exemption trial, the ACURATE platform was withdrawn from the market in May 2025, mainly due to unproportional regulatory demands by the European notified body. However, although the device is no longer available for clinical use, reporting long-term clinical and echocardiographic data remains clinically relevant given approximately 100,000 implantations worldwide. In particular, insights into the timing and predominant mode of failure would be valuable for the ongoing management of patients treated with this platform.

This study aimed to analyze long-term outcomes and echocardiographic data among ACURATE neo recipients to determine rates of SVD and BVF.

## Methods

### Study Cohort

Consecutive patients undergoing transfemoral TAVI for severe native aortic stenosis from 4 centers (Kerckhoff Heart Center, Bad Nauheim, Justus-Liebig University of Giessen, Giessen, Germany; Heart Center Lucerne, Lucerne, Switzerland; Instituto Dante Pazzanese de Cardiologia, São Paulo, Brazil) using the ACURATE neo between 2012 and 2018 (n = 980) were screened for study inclusion.

The characteristics and implantation technique of the ACURATE neo have been described previously.^3^ All patients were discussed within the Heart Team of each center in adherence to existing guidelines.^6^

**Figure 1:**
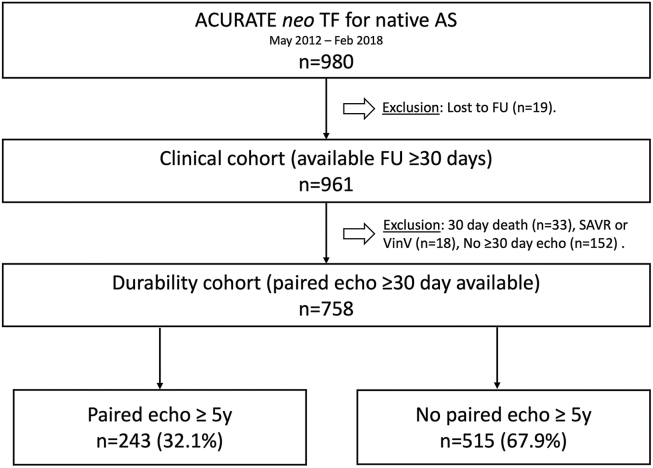
**Study flow chart.** Abbreviations: AS, aortic stenosis; FU, follow-up, SAVR, structural valve deterioration; TF, transfemoral; VinV, valve-in-valve implantation.

The flowchart in Figure 1 shows the different subsets of patients with specific inclusion and exclusion criteria according to the endpoint. All patients with available vital status information were included in the survival analysis (overall cohort). For the durability analysis, patients with 30-day mortality, with valve-in-valve implantation or surgical aortic valve replacement within 30 days, or without echocardiographic FU beyond 30 days were excluded, resulting in a durability cohort of 758 patients.

At Kerckhoff Heart Center, patients who were alive without recent FU echocardiography were contacted and asked for participation in the study with written informed consent. Those who agreed to participate were scheduled either for an outpatient visit in our hospital or, if unable to travel, a home visit to obtain transthoracic echocardiography examinations. Patients that declined study participation were asked for written permission to request medical records of the most recent examination (which included echocardiograms, patient history, and medication) by their cardiologist or family doctor. At the remaining centers, the FU visits and echocardiography were part of the clinical routine and were analyzed retrospectively.

Patients who could not be contacted and of whom the alive status could not be determined were declared as lost-to-FU. Among patients who died, we attempted to obtain data on the cause of death and records on the most recent echocardiography before death by contacting relatives, referring physicians, or medical reports. The census of FU was February 2024.

Baseline data including demographics, comorbidities, risk scores, and echocardiography results were recorded and consolidated in a joint database. Any inconsistencies of the data were resolved by direct communication with the investigator of each participating center. The study was conducted in adherence to the Declaration of Helsinki and was approved by the local ethics committees.

### Imaging Workup

Echocardiography assessment included baseline transthoracic echocardiography, post-TAVI (either in-hospital before discharge or at 30-day FU), and the last available FU echocardiography. Long-term valve performance was assessed among patients with available paired FU echocardiography beyond 5 years.

In addition to standard measurements of aortic root dimensions on computed tomography, the cover index (%) was determined as 100 × ([prosthesis size (mm) – perimeter derived annulus diameter (mm)]/prosthesis size [mm]). The aortic valve calcium score was measured according to the Agatston method using noncontrast-enhanced multidetector computed tomography (scans).

### Outcomes of Interest

The primary outcome was the cumulative incidence of late (>30 days after the index procedure) BVF, encompassing (1) valve-related death, (2) severe hemodynamic SVD, or (3) aortic valve reintervention. Valve-related death was assumed when the date of death was within 30 days following either aortic valve reintervention or diagnosis of SVD. Coprimary outcomes were valve performance beyond 5-year FU and the cumulative incidence of SVD. Moderate hemodynamic SVD was defined as (1) the mean gradient ≥20 and < 40 mmHg and/or ≥10 and < 20 mmHg change from baseline (before discharge or within 30 days of valve implantation) and/or (2) moderate new or worsening (>1+/4+) intraprosthetic aortic regurgitation (AR). Severe hemodynamic SVD was defined as (1) the mean gradient ≥40 mmHg and/or ≥20 mmHg change from baseline (before discharge or within 30 days of valve implantation) and/or (ii) severe new or worsening (>1+/4+) intraprosthetic AR. Morphological SVD was defined as abnormalities of the following domains: leaflet integrity (i.e., torn or flail causing intraframe regurgitation), leaflet structure (i.e., pathological thickening and/or calcification causing valvular stenosis or central regurgitation), leaflet function (i.e., impaired mobility resulting in stenosis and/or central regurgitation), and strut/frame (i.e., fracture or failure).^7^ Secondary outcomes were time-to-event rates of all-cause mortality, stroke, and hospitalization (valve-related or worsening heart failure) in the total cohort.

### Statistical Analysis

Continuous data are presented as median and interquartile range (IQR). Comparison of groups was performed using the Mann-Whitney *U* test and the Chi-squared or Fisher exact test, as appropriate. The reverse Kaplan–Meier method was used to calculate the median FU time. The estimates of late BVF and SVD over time were assessed with the Kaplan–Meier method (actuarial analysis) and the cumulative incidence method according to Aalen–Johansen (actual analysis), accounting for the competing risk of death. In this elderly population with high competing mortality, cumulative incidence estimates more appropriately reflect the risk of experiencing valve-related events and avoid overestimation inherent to the Kaplan–Meier method, which treats death as a censoring event. Furthermore, incidence rates of BVF and SVD per 100 patient-years were calculated. Cox regression analyses were performed to determine predictors of all-cause mortality beyond 30 days, SVD, and late BVF; for this purpose, all variables with *p* values <0.10 in the univariate analysis or that were deemed clinically relevant were entered into the multivariable analysis. Proportional hazards assumption was tested using Schoenfeld residuals, and collinearity was assessed using variance inflation factors. Continuous variables were assessed for linearity using Martingale residuals and visual inspection of LOWESS-smoothed plots (locally weighted scatterplot smoothing). For all analyses, a 2-sided *p* value <0.05 was considered to indicate statistical significance. All analyses were performed with STATA IC (version 18.0 BE; StataCorp LCC, College Station, Texas).

## Results

### Study Cohort

The study flowchart is shown in Figure 1. The median time to death or last FU in the overall cohort was 5.0 years (IQR 2.3–6.4) with a maximum FU time of 11.3 years. At the time of the census, there were 409 survivors (42.6%) with a median FU time of 6.2 years (IQR 5.2–7.3). The number of patients with available paired FU echocardiography beyond 30 days was 758 (78.9%), whereas 243 (25.3%) patients had a FU echo beyond 5 years with a median FU time of 6.4 years (IQR 5.9–7.4) and a maximal FU time of 11.0 years. Baseline characteristics are summarized in Table 1. The median age was 82.0 years (IQR 78.8–85.4), 61.3% were females, and the median EuroScore II was 3.3% (IQR .3–5.6). Procedural details and in-hospital outcomes are provided in Table 2.

**Table 1:** Baseline characteristics

Variable	Overall cohort n = 961	Durability cohort n = 758	Excluded cohort n = 203	*p*
Age, y	82.0 [78.8–85.4]	81.7 [78.5–84.9]	83.7 [80.2–87.1]	<0.001
Female sex	589 (61.3%)	461 (60.8%)	128 (63.1%)	0.561
Body mass index, kg/m^2^	26.9 [24.1–30.4]	27.2 [24.3–30.5]	25.8 [23.7–9.0]	0.003
EuroSCORE II, %	3.3 [2.3–5.6]	3.3 [2.2–5.6]	3.4 [2.5–5.6]	0.282
eGFR, ml/min/1.73 m^2^	60.0 [43.0–80.0]	60.4 [44.0–80.0]	57.0 [38.0–79.0]	0.129
Coronary artery disease	549 (57.1%)	435 (57.4%)	114 (56.2%)	0.753
Prior stroke	107 (11.1%)	80 (10.6%)	27 (13.3%)	0.269
Atrial fibrillation	347 (36.2%)	260 (34.4%)	87 (42.9%)	0.027
Prior PPI	100 (10.4%)	76 (10.0%)	24 (11.8%)	0.457
COPD	182 (18.9%)	143 (18.9%)	39 (19.2%)	0.911
Ejection fraction, %	65 [55–65]	65 [55–65]	64 [55–65]	0.288
Mean gradient, mmHg	42 [32–53]	43 [33–53]	41 [29–53]	0.050
AVA, cm^2^	0.7 [0.6–0.8]	0.7 [0.6–0.8]	0.7 [0.6–0.8]	0.306
Perimeter-derived annulus, mm	23.8 [22.6–25.1]	23.8 [22.6–25.0]	23.9 [22.6–25.5]	0.322
Cover index, %	4.8 [2.8–7.2]	4.9 [2.9–7.3]	4.5 [2.2–7.1]	0.065
AVCS, AU	2252 [1570–3056]	2228 [1559–3037]	2292 [1628–3193]	0.354

Values denote median [IQR] or n (%).

Abbreviations: AU, arbitrary unit; AVA, aortic valve area; AVCS, aortic valve calcium score; COPD, chronic obstructive pulmonary disease; eGFR, estimated glomerular filtration rate; IQR, interquartile range; PPI, permanent pacemaker implantation.

**Table 2:** Procedural outcomes and complications

Variable	Overall cohort n = 961	Durability cohort n = 758	Excluded cohort n = 203	*p*
Procedural data				
Prosthesis size				0.093
S, 23 mm	244 (25.4%)	104 (24.3%)	60 (29.6%)	
M, 25 mm	408 (42.5%)	335 (44.2%)	73 (36.0%)	
L, 27 mm	309 (32.2%)	239 (31.5%)	70 (34.5%)	
Procedural duration, min	41 [32–56]	40 [32–56]	42 [34–57]	0.266
Fluoroscopy time, min	9.6 [7.4–12.5]	9.3 [7.2–12.0]	10.4 [7.6–14.5]	0.001
Contrast agent, ml	94 [70–120]	95 [72–120]	89 [70–120]	0.433
Predilatation	768 (79.9%)	609 (80.3%)	159 (78.3%)	0.524
Postdilatation	346 (36.0%)	270 (35.6%)	76 (37.4%)	0.632
Ejection fraction post, %	65.0 [60.0–65.0]	65.0 [60.0–65.0]	65.0 [55.0–65.0]	0.455
Mean gradient post, mmHg	8 [5–11]	8 [6–11]	7 [5–10]	0.005
EOA post, cm^2^	1.7 [1.5–2.0]	1.7 [1.5–2.0]	1.7 [1.4–1.9]	0.390
Implantation depth NCC, mm	5.0 [4.0–6.0]	5.0 [4.0–6.0]	5.0 [4.0–7.0]	0.679
Technical success (VARC-3)	899 (93.5%)	732 (96.6%)	167 (82.3%)	<0.001
Device success (VARC-3)	799 (83.1%)	674 (88.9%)	125 (61.6%)	<0.001
Complications				
PVL post-TAVI				0.004
None/trace	309 (32.5%)	237 (31.3%)	72 (37.3%)	
Mild	601 (63.2%)	495 (65.3%)	106 (54.9%)	
Moderate	41 (4.3%)	26 (3.4%)	15 (7.8%)	
Severe PPM	24/520 (4.6%)	21/408 (5.1%)	3/112 (2.7%)	0.270
Major cardiac structural complication	23 (2.4%)	10 (1.3%)	13 (6.4%)	<0.001
Major vascular complication	99 (10.5%)	68 (9.0%)	31 (15.3%)	0.009
Bleeding BARC 2-4	159 (16.5%)	103 (13.6%)	56 (27.6%)	<0.001
Any stroke	25 (2.6%)	11 (1.5%)	14 (6.9%)	<0.001
Delirium	43/876 (4.9%)	20/678 (2.9%)	23/198 (11.6%)	<0.001
Acute kidney injury St. 2-4	71/808 (8.8%)	40/623 (6.4%)	31/185 (16.8%)	<0.001
Pacemaker implantation	85 (8.8%)	56 (7.4%)	29 (14.3%)	0.002

Values denote median [IQR] or n (%).

Abbreviations: BARC, Bleeding Academic Research Consortium; EOA, effective orifice area; IQR, interquartile range; NCC, noncoronary cusp; PPM, prosthesis-patient mismatch; PVL, paravalvular leakage; TAVI, transcatheter aortic valve implantation; VARC, Valve Academic Research Consortium.

### Long-Term Survival

**Figure 2:**
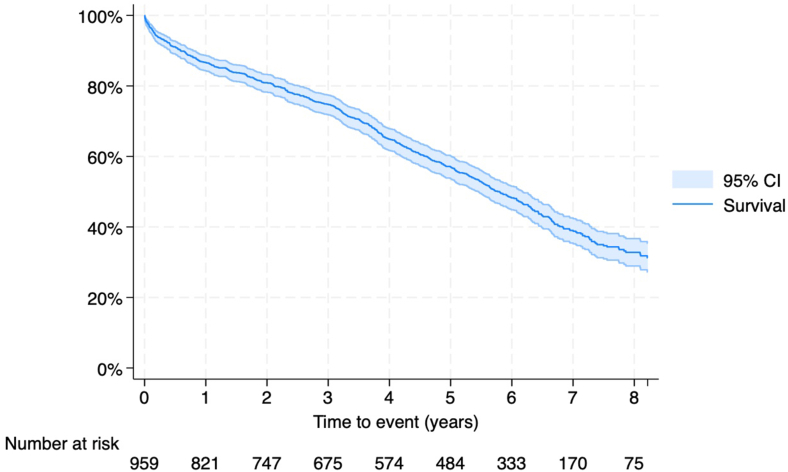
Survival estimates in the overall cohort.

In the overall cohort, the Kaplan–Meier estimates of all-cause mortality at 1, 5, and 8 years were 13.6% (95% CI 11.6–15.9), 43.1% (95% CI 39.9–46.4), and 67.3% (95% CI 63.4–71.1), respectively (Figure 2). Higher age, atrial fibrillation, renal failure, lower mean aortic valve gradient at baseline and postprocedure, acute kidney injury, and delirium were independent predictors of all-cause mortality beyond 30 days (Table 3).

**Table 3:** Cox regression analysis for all-cause mortality beyond 30 days

Covariate	Hazard ratio (95% CI)	*p*	Hazard ratio_adj_ (95% CI)	*p*
Age, per year	1.05 (1.03–1.07)	**<0.001**	1.03 (1.01–1.05)	**<0.001**
Female sex	1.04 (0.87–1.25)	0.629		
Coronary artery disease	1.11 (0.94–1.33)	0.211		
Atrial fibrillation	1.80 (1.51–2.15)	**<0.001**	1.22 (1.00–1.50)	**0.049**
eGFR, per ml/min	0.99 (0.98–0.99)	**<0.001**	0.99 (0.99–0.99)	**0.007**
Pmean_pre_, per mmHg	0.98 (0.97–0.99)	**<0.001**	0.99 (0.98–0.99)	**0.041**
AVCS, per AU	0.99 (0.99–1.00)	0.796		
EuroSCORE II, per %	1.03 (1.01–1.05)	**0.001**	1.01 (0.98–1.03)	0.517
New LBBB	0.74 (0.43–1.26)	0.270		
PPI	1.56 (1.19–2.05)	**0.001**	1.33 (0.99–1.77)	0.053
Bleeding BARC 2-4	1.40 (1.12–1.75)	**0.003**	1.12 (0.87–1.43)	0.371
Major vasc complication	1.27 (0.96–1.68)	0.087		
Stroke	1.37 (0.75–2.49)	0.304	0.94 (0.46–1.90)	0.858
Delirium	2.32 (1.60–3.36)	**<0.001**	1.80 (1.20–2.71)	**0.004**
AKI St. 2-4	1.09 (0.94–1.84)	0.110	1.48 (1.02–2.14)	**0.036**
Pmean post	0.93 (0.91–0.95)	**<0.001**	0.95 (0.93–0.97)	**<0.001**
Severe PPM	1.13 (0.68–1.86)	0.639		
PVL ≥ moderate	1.30 (0.89–1.91)	0.167	1.11 (0.74–1.68)	0.594

Significant *p*-values are highlighted in bold.

Abbreviations: AKI, acute kidney injury; AU, arbitrary unit; AVCS, aortic valve calcium score; BARC, Bleeding Academic Research Consortium; eGFR, estimated glomerular filtration rate; LBBB, left bundle branch block; Pmean, mean aortic gradient; PPI, permanent pacemaker implantation; PPM, prosthesis-patient mismatch; PVL, paravalvular leakage; vasc, vascular.

### Valve Performance

**Figure 3:**
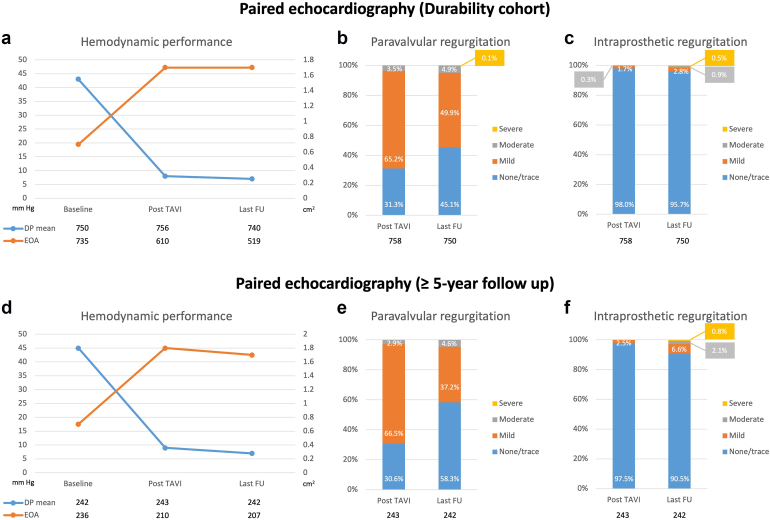
**Long-term valve performance.** (A to C) Valve performance at last FU in the durability cohort (n = 758) and (D to F) the subset of patients with echocardiography FU beyond 5 years (n = 243). Abbreviations: DP mean, mean aortic gradient; EOA, effective orifice area; FU, follow-up; TAVI, transcatheter aortic valve implantation.

The valve performance in the SVD cohort and in the subset of patients with echocardiography FU beyond 5 years is shown in Figure 3. In the latter group, the mean aortic valve gradients and effective orifice area were 45 mmHg (IQR 37–54) and 0.7 cm^2^ (IQR 0.6–0.8) at baseline, 9 mmHg (IQR 7–13) and 1.8 cm^2^ (1.5–2.0) post-TAVI, and 6 mmHg (IQR 4–8) and 1.7 cm^2^ (IQR 1.4–2.0) at the final FU echocardiography. Paravalvular leakage (PVL) ≥ moderate was 2.8% (7/243) post-TAVI and 4.6% (11/242) at long-term FU. Of the 7 cases with PVL ≥ moderate post-TAVI, 4 were classified as PVL ≤ mild at final FU echo. Meanwhile, of the 11 cases with PVL ≥ moderate at final FU echocardiography, 8 had been classified as ≤ mild at post-TAVI. Supplemental Table 1 compares patients with echocardiography FU ≥ 5 years versus those without within the SVD cohort.

### Structural Valve Deterioration and Bioprosthetic Valve Failure

**Figure 4:**
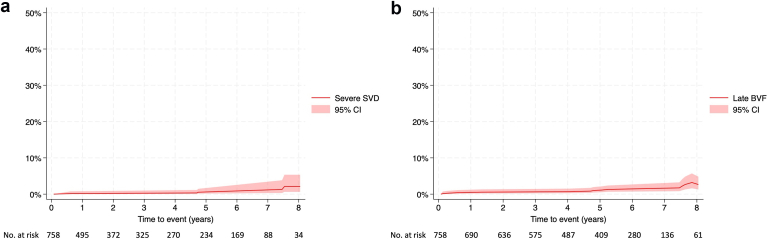
**Durability outcomes:cumulative incidence of (A) severe SVD and (B) late BVF.** Abbreviations: BVF, bioprosthetic valve failure; SVD, structural valve deterioration.

Estimates of late BVF and SVD are shown in Table 4 and Figure 4. The cumulative incidence of moderate SVD was 3.5% (95% CI 1.5–6.7). The cumulative incidence of severe SVD was 0.5% (95% CI 0.01–0.1) and 2.1% (95% CI 0.6–5.3) at 5 and 8 years, respectively. During 2313.9 patient-years of FU, the incidence rate of SVD was 64.8 (95% CI 39.1–107.5) per 100 patient-years. The cumulative incidence of late BVF was 0.9% (95% CI 0.04–0.2) and 3.2% (95% CI 1.6–5.7) at 5 and 8 years, respectively. During 3639.7 patient-years of FU, the incidence rate of late BVF was 35.7 (95% CI 20.7–61.5) per 100 patient-years.

**Table 4:** Durability outcomes

Outcome (n = 758)	Actual analysis 8-y Cumulative incidence	Actuarial analysis 8-y Kaplan–Meier	Incidence rate per 100 patient-y
Moderate SVD, %	3.5 (1.5–6.7)	7.6 (3.5–15.9)	43.2 (23.3–80.3)∗
Severe SVD, %	2.1 (0.6–5.3)	4.6 (1.5–13.6)	21.6 (8.9–51.9)∗
BVF, %	3.2 (5.7–1.6)	6.5 (3.1–13.3)	46.7 (20.7–61.5)†

Values are presented as % (95% CI).

The overall median FU time in the durability cohort was 5.5 y (IQR 3.4–6.7) and 6.2 y (IQR 5.4–7.3) among survivors.

Abbreviations: BVF, bioprosthetic valve failure; FU, follow-up; IQR, interquartile range; SVD, structural valve deterioration.

∗ 2313.9 patient-year.

† 3639.7 patient-year.

Table 5 lists all BVF and SVD cases including the failure mode, time to failure, and vital status. Tables 6 and 7 show Cox regression analyses for any SVD and BVF. Independent predictors of any SVD were left ventricular ejection fraction at baseline (adjusted hazard ratio 1.13 [95% CI 1.02; 1.25]; *p* = 0.019) and PVL ≥ moderate at discharge (adjusted hazard ratio 7.27 [95% CI 1.42; 37.12]; *p* = 0.017). Independent predictors of BVF were younger age (adjusted hazard ratio per year 0.91 [95% CI 0.84; 0.98]; *p* = 0.021) and PVL ≥ moderate at discharge (adjusted hazard ratio 7.98 [95% CI 2.18; 29.22]; *p* = 0.002).

**Table 5:** Overview of cases with BVF or SVD

Case	BVF	SVD	Failure mode/reason for intervention	Time to BVF or SVD, d	Vital status at last FU	Time to last FU, d
1	Redo-TAVI	Severe SVD	Valvular AR	2752	Alive	3129
2	Redo-TAVI	Severe SVD	Valvular AR	1751	Dead	3347
3		Moderate SVD	Valvular AR	2672	Alive	2921
4	Redo-TAVI	Moderate SVD	Valvular AR	2787	Dead	2830
5	Redo-TAVI	Severe SVD	Valvular AR	1718	Alive	2837
6	SAVR		Endocarditis	2864	Alive	2864
7		Moderate SVD	High gradient	2012	Alive	2012
8		Moderate SVD	Valvular AR	2624	Dead	2655
9	Severe SVD	Severe SVD	High gradient	2723	Alive	2723
10	Severe SVD	Severe SVD	Valvular AR	223	Dead	667
11		Moderate SVD	High gradient	450	Dead	2064
12	SAVR Valve-related death		Endocarditis	50	Dead	51
13		Moderate SVD	High gradient	426	Alive	1539
14	Redo-TAVI		PVL	114	Alive	2447
15		Moderate SVD	Valvular AR High gradient	2226	Alive	2226
16	Redo-TAVI		Hemolysis	481	Alive	2205
17	Redo-TAVI	Moderate SVD	Valvular AR	1485	Alive	2116
18		Moderate SVD	High gradient	1090	Alive	1090
19	Redo-TAVI	Moderate SVD	Valvular AR High gradient	1913	Dead	2157
20	Valve-related death		Leaflet thrombosis	1850	Dead	1850

Abbreviations: AR, aortic regurgitation; BVF, bioprosthetic valve failure; FU, follow-up; PVL, paravalvular leakage; SAVR, surgical aortic valve replacement; SVD, structural valve deterioration; TAVI, transcatheter aortic valve implantation.

**Table 6:** Cox regression analysis for any SVD

Covariate	Hazard ratio (95% CI)	*p*	Hazard ratio_adj_ (95% CI)	*p*
Age, per year	0.95 (0.86–1.03)	0.211	0.93 (0.85–1.01)	0.104
Female sex	0.67 (0.23–1.98)	0.476		
Hypertension	0.00 (0.00–0.00)	1.000		
Diabetes	0.99 (0.34–2.91)	0.995		
Hyperlipidemia	0.53 (0.16–1.76)	0.303		
eGFR, per ml/min	1.00 (0.99–1.02)	0.319		
Pmean_pre_, per mmHg	1.1 (0.98–1.05)	0.294		
AVCS, per AU	0.99 (0.99–1.00)	0.820		
Cover index, per %	0.88 (0.76–1.04)	0.140	0.88 (0.75–1.03)	0.118
Predilatation	0.50 (0.16–1.60)	0.244		
Postdilatation	1.43 (0.52–3.97)	0.489		
LVEF post, per %	1.10 (1.00–1.20)	**0.044**	1.13 (1.02–1.25)	**0.019**
Pmean post	1.03 (0.92–1.14)	0.618		
Moderate or severe PPM	0.70 (0.14–3.51)	0.672		
PVL ≥ moderate	4.25 (0.96–18.91)	0.057	7.27 (1.42–37.12)	**0.017**

Significant *p*-values are highlighted in bold.

Abbreviations: AU, arbitrary unit; AVCS, aortic valve calcium score; eGFR, estimated glomerular filtration rate; LVEF, left ventricular ejection fraction; Pmean, mean aortic gradient; PPM, prosthesis-patient mismatch; PVL, paravalvular leakage; SVD, structural valve deterioration.

**Table 7:** Cox regression analysis for BVF

Covariate	Hazard ratio (95% CI)	*p*	Hazard ratio_adj_(95% CI)	*p*
Age, per year	0.92 (0.85–1.00)	0.058	0.91 (0.84–0.98)	**0.021**
Female sex	1.83 (0.68–4.87)	0.228		
Hypertension	0.63 (0.14–2.79)	0.545		
Diabetes	0.73 (0.23–2.27)	0.592		
Hyperlipidemia	1.83 (0.56–6.01)	0.318		
eGFR, per ml/min	0.99 (0.98–1.01)	0.631		
Pmean_pre_, per mmHg	0.98 (0.95–1.02)	0.395		
AVCS, per AU	1.00 (0.99–1.00)	0.836		
Predilatation	0.39 (0.12–1.33)	0.134		
Postdilatation	1.53 (0.51–4.58)	0.443		
Pmean post	0.96 (0.85–1.08)	0.482		
Moderate or severe PPM	0.59 (0.12–2.84)	0.511		
PVL ≥ moderate	5.96 (1.69–21.01)	**0.005**	7.98 (2.18–29.22)	**0.002**

Significant *p*-values are highlighted in bold.

Abbreviations: AU, arbitrary unit; AVCS, aortic valve calcium score; BVF, bioprosthetic valve failure; eGFR, estimated glomerular filtration rate; Pmean, mean transaortic gradient; PPM, prosthesis-patient mismatch; PVL, paravalvular leakage.

## Discussion

We present the first long-term outcomes including echocardiography after transfemoral TAVI using the ACURATE neo THV from 4 international centers. The main findings of the present study are as follows.

(i) The Kaplan–Meier estimates of all-cause mortality at 5 and 8 years were 43.1 and 67.3%, respectively.
(ii) At 8 years, the cumulative incidence was 3.5% for moderate SVD, 2.1% for severe SVD, and 3.2% for BVF.
(iii) Long-term valve performance after a median of 6.4 years was preserved.

### Long-Term Mortality

The all-cause mortality we observed in the present study is consistent to previous reports on long-term survival in intermediate-to high-risk TAVI cohorts. In the Neo2 CE-mark study, 5-year all-cause mortality was 44%,^8^ whereas in a meta-analysis by Shimamura et al.^9^ involving various early-generation prosthesis types, 5-year all-cause mortality was 53%. Estimates of 8-year all-cause mortality in intermediate-to high-risk populations vary between 70 and 80%.^10^, ^11^, ^12^, ^13^ The fact that the 8-year all-cause mortality is in a lower range is notable, given that the Achilles’ heel of the ACURATE neo was PVL ≥ moderate, which has been reported at approximately 10% in the randomized SCOPE I and II trials and in some observational studies.^14^^,^^15^ In the SCOPE I trial, the failure of the ACURATE neo to meet noninferiority versus the balloon-expandable SAPIEN 3 prosthesis was mainly driven by higher rates of acute kidney injury and PVL ≥ moderate (9 vs. 3%).^14^ Nevertheless, at 3-year FU of the SCOPE I study, all-cause mortality was similar between the 2 groups, even showing a crossing of the survival curves.^16^ Likewise, in the present ACURATE neo cohort, long-term mortality was not majorly affected by PVL ≥ moderate, which is corroborated by our finding that PVL ≥ moderate was not associated with long-term all-cause mortality in the Cox regression analysis (Table 4). Indeed, similar observations have been made in previous investigations with other THVs.^10^^,^^11^^,^^17^

The finding that lower post-procedural mean transvalvular gradients were independently associated with all-cause mortality over time seems to be counterintuitive but may be explained by low-flow aortic stenosis indicating left ventricular damage.

### Long-Term Valve Performance

In the subset of patients with long-term echocardiography FU, hemodynamic performance was preserved over a median FU time of 6.4 years (Figure 3). Interestingly, there was a slight increase in PVL ≥ moderate from 2.9 to 4.6% but a decrease in mild PVL from 66.5 to 37.2%. Regarding valvular AR, the rate of at least mild regurgitation increased over time from 2.5% to almost 10%. However, most cases were mild and not clinically relevant; only 3% had moderate or severe valvular AR.

### Durability Data

As this is the first report of THV durability outcomes for the ACURATE platform, no previous comparative data are available. The cumulative incidence of moderate SVD (3.5%) and severe SVD (2.1%) and that of BVF (3.2%) of the ACURATE neo THV at 8 years is in the range of durability data of other THV types beyond 5 years, although most existing studies are limited to retrospective analyses predominantly from single centers with relatively small sample sizes and incomplete echocardiography assessments. Another major limitation that may preclude comparability is that, in some studies, the denominators of SVD and BVF analyses may not have been well defined. As the definition of both SVD and BVF requires echocardiography, studies with incomplete echocardiography FU may have overestimated the denominator by inclusion of patients without recent echocardiography data.^10^, ^11^, ^12^, ^13^ The only durability report from a randomized trial originates from the NOTION study, which compared TAVI using the self-expanding CoreValve THV versus surgical aortic valve replacement, although the TAVI arm included only 145 patients. At 8 years, the cumulative incidence of SVD in the TAVI arm was 13.9%, and the cumulative incidence of BVF was 8.7%.^17^ In a meta-analysis by Shimamura et al.^9^, freedom from SVD was 95.5% at 5 years and 15.1% at 8 years. Several other retrospective registries analyzing durability data beyond 5-year FU report inconsistent BVF rates between 4.6 and 18.0% using actuarial analysis^10^^,^^12^^,^^13^ and between 2.5 and 8.7% using actual analysis,^10^^,^^12^^,^^13^^,^^17^ whereas rates of SVD vary according to the definition and applied method (moderate SVD: 3.6 to 14.9%; severe SVD: 2.4 to 5.9%).^12^^,^^13^^,^^17^

The finding that PVL ≥ moderate was an independent predictor of both BVF and SVD (Tables 6 and 7) is difficult to explain, but it likely reflects an indirect pathophysiologically plausible association, as PVL may adversely affect hemodynamic flow and increase turbulence and stress on the leaflets and thereby accelerate valve degeneration. Regarding BVF, redo-TAVI for relevant PVL may additionally contribute to this observed association.

### Failure Mode

The primary failure mode of the ACURATE neo THV seems to be valvular AR. In our series, two-thirds of SVD cases exhibited valvular AR (Table 5), and valve reinterventions were commonly performed for valvular AR. Even though a direct comparison is not appropriate, previous data with other THV types suggest a lower proportion of valvular AR as failure mode. In the 8-year FU of the NOTION trial, the rate of SVD after TAVI with the CoreValve prosthesis was 13.9%, of which 3.7% had valvular AR.^17^ Of 10 cases of SVD in another CoreValve long-term FU series, 4 had relevant valvular AR and another 4 had both valvular AR and stenosis as mode of failure.^13^ Long-term FU data from a cohort with both self-expanding and balloon-expandable THV showed that among 7 cases with SVD beyond 30 days, 4 had stenosis, 2 had relevant valvular AR, and 1 had mixed mode of failure.^12^

## Limitations

The present study represents a retrospective multicenter cohort analysis with inherent limitations. FU strategies, including the timing and availability of echocardiographic examinations, differed between participating centers and were largely driven by routine clinical practice. Consequently, serial echocardiography at regular annual intervals was available in only a minority of patients, precluding a detailed assessment of gradual temporal changes in valve performance. Long-term echocardiographic FU beyond 5 years was available only in a subset of surviving patients, introducing potential survivor bias and limiting the generalizability of long-term valve performance findings.

Echocardiographic assessments were performed locally without adjudication by an independent core laboratory. Although core laboratory adjudication improves standardization and reduces interobserver variability, it does not eliminate measurement uncertainty. Given that durability endpoints were primarily driven by predefined hemodynamic criteria or clinical valve failure, any potential misclassification is unlikely to have materially affected the main conclusions. Regression analyses must be considered exploratory and hypothesis-generating. Finally, given the advanced age and risk profile of the study population, the findings should not be extrapolated to younger or lower-risk patients or to contemporary THV platforms.

## Conclusions

In this multicenter observational cohort of patients treated with the ACURATE neo platform with extended FU, survival rate was 32.7%, and the cumulative incidence for severe SVD (2.1%) and BVF (3.2%) through 8 years was low. Within the limitations of the present analysis, these findings provide long-term durability estimates in an elderly, real-world population and should be interpreted in this clinical context.

## Funding

This work received financial support from 10.13039/100008497Boston Scientific.

## Disclosure Statement

Won-Keun Kim reports receipt of proctor/speaker honoraria from Abbott, Anteris, Boston Scientific, Cardiawave, Edwards Lifesciences, HID Imaging, Jenavalve, Medtronic, Meril Life Sciences, and P & F. Helge Möllmann reports receipt of honoraria or consultation fees from Abbott, Biotronik, Boston Scientific, Edwards Lifesciences, Medtronic, and SMT. Matthias Renker reports receipt of proctor honoraria from Boston Scientific and Medtronic. Efstratios Charitos reports receipt of proctor honoraria from Boston Scientific. Yeong-Hoon Choi reports receipt of speaker/proctor fees from Edwards Lifesciences, Cytosorbents, and Getinge. Stefan Toggweiler reports receipt of honoraria from Medtronic, Boston Scientific, Biosensors, Edwards Lifesciences, Hi-D Imaging, Abbott Vascular, Medira, Shockwave, Teleflex, atHeart Medical, Cardiac Dimensions, Polares Medical, Amarin, Sanofi, AstraZeneca, ReCor Medical, Daiichi Sankyo, Bayer, and Armira; reports receipt of institutional research grants from Edwards Lifesciences, Abbott Vascular, Boston Scientific, Fumedica, Novartis, Boehringer Ingelheim, and Polares Medical; is the founder and owner of CardiaNova GmbH; holds equity in Hi-D Imaging; and has filed a patent application for a balloon catheter (10 2015 114 609.8).

The other authors had no conflicts to declare.
