# Supplementary material for: Long-Term Outcomes of a Self-Expanding Transcatheter Heart Valve
Source: Struct Heart. 2026 Apr 4;10(6):100841. doi: 10.1016/j.shj.2026.100841 (PMC13316482; doi:10.1016/j.shj.2026.100841)
Supplement: Supplementary Material [file mmc1.docx]

**Long-term outcomes of a self-expanding transcatheter heart valve**

**Supplemental Table 1: Comparison of patients with extended echocardiography follow up versus those without**

| Variable | No Echo FU ≥ 5y  n=515 | Echo FU ≥ 5y  n=243 | p |
| --- | --- | --- | --- |
| Age, years | 82.0 [78.8–85.5] | 80.6 [77.9–84.0] | <0.001 |
| Female sex | 329 (63.9%) | 132 (54.3%) | 0.012 |
| Body mass index, kg/m^2^ | 27.1 [24.2–30.5] | 27.4 [24.7–30.7] | 0.174 |
| EuroSCORE II, % | 3.3 [2.3–5.6] | 3.1 [2.1–5.5] | 0.176 |
| eGFR, ml/min/1.73 m^2^ | 56.9 [41.0–79.0] | 67.0 [48.8–82.0] | <0.001 |
| Coronary artery disease | 288 (55.9%) | 147 (60.5%) | 0.235 |
| Prior stroke | 56 (10.9%) | 24 (9.9%) | 0.677 |
| Atrial fibrillation | 210 (40.9%) | 50 (20.7%) | <0.001 |
| Prior PPI | 52 (10.1%) | 24 (9.9%) | 0.925 |
| COPD | 104 (20.2%) | 39 (16.1%) | 0.173 |
| Ejection fraction, % | 65 [55–65] | 65 [55–65] | 0.266 |
| Mean gradient, mmHg | 42 [32–53] | 45 [37–54] | 0.003 |
| AVA, cm^2^ | 0.7 [0.6–0.8] | 0.7 [0.6–0.8] | 0.569 |
| Perimeter-derived annulus, mm | 23.8 [22.8–25.1] | 23.7 [22.5–25.0] | 0.137 |
| Cover index, % | 4.9 [2.8–7.1] | 4.8 [2.9–7.9] | 0.317 |
| AVCS, AU | 2166 [1491–2986] | 2437 [1645–3104] | 0.084 |
| Center  A  B  C  D | 297 (57.7%)  113 (21.9%)  26 (5.1%)  79 (15.3%) | 149 (61.3%)  22 (9.1%)  54 (22.2%)  18 (7.4%) | <0.001 |

Values denote median [interquartile range] or n (%).

Abbreviations: AV = aortic valve, AVA = aortic valve area, AVCS = aortic valve calcium score, eGFR = estimated glomerular filtration rate, Echo FU≥5y = extended echocardiography follow up beyond 5 years

**Supplemental Table 2: Distribution of annual echocardiography follow ups**

| Echocardiography FU | Number (%) | Cumulative number (%) |
| --- | --- | --- |
| 1-year | 271 (35.8%) | 271 (35.8%) |
| 2-year | 115 (15.2%) | 386 (50.9%) |
| 3-year | 49 (6.5%) | 435 (57.4%) |
| 4-year | 53 (7.0%) | 488 (64.4%) |
| 5-year | 35 (4.6%) | 523 (69.0%) |
| 6-year | 65 (8.6%) | 588 (77.6%) |
| 7-year | 80 (10.6%) | 668 (88.1%) |
| 8-year | 54 (7.1%) | 722 (95.3%) |
| 9-year | 30 (4.0%) | 752 (99.2%) |
| 10-year | 4 (0.5%) | 756 (99.7%) |
| 11-year | 2 (0.3%) | 758 (100.0%) |
